# Monogenic hyperinsulinemic hypoglycemia: current insights into the pathogenesis and management

**DOI:** 10.1186/1687-9856-2013-3

**Published:** 2013-02-06

**Authors:** Katherine Lord, Diva D De León

**Affiliations:** 1 Division of Endocrinology and Diabetes, The Children’s Hospital of Philadelphia, 3615 Civic Center Boulevard, Abramson Research Center Room 802A, Philadelphia, PA, 19104, USA; 2 Department of Pediatrics, Perelman School of Medicine at the University of Pennsylvania, Philadelphia, PA, USA

**Keywords:** Beta-cell, Neonatal hypoglycemia, Insulin, K_ATP_ channel, ^18^ F-DOPA PET

## Abstract

Hyperinsulinism (HI) is the leading cause of persistent hypoglycemia in children, which if unrecognized may lead to development delays and permanent neurologic damage. Prompt recognition and appropriate treatment of HI are essential to avoid these sequelae. Major advances have been made over the past two decades in understanding the molecular basis of hyperinsulinism and mutations in nine genes are currently known to cause HI. Inactivating K_ATP_ channel mutations cause the most common and severe type of HI, which occurs in both a focal and a diffuse form. Activating mutations of glutamate dehydrogenase (GDH) lead to hyperinsulinism/hyperammonemia syndrome, while activating mutations of glucokinase (GK), the “glucose sensor” of the beta cell, causes hyperinsulinism with a variable clinical phenotype. More recently identified genetic causes include mutations in the genes encoding short-chain 3-hydroxyacyl-CoA (SCHAD), uncoupling protein 2 (UCP2), hepatocyte nuclear factor 4-alpha (HNF-4α), hepatocyte nuclear factor 1-alpha (HNF-1α), and monocarboyxlate transporter 1 (MCT-1), which results in a very rare form of HI triggered by exercise. For a timely diagnosis, a critical sample and a glucagon stimulation test should be done when plasma glucose is < 50 mg/dL. A failure to respond to a trial of diazoxide, a K_ATP_ channel agonist, suggests a K_ATP_ defect, which frequently requires pancreatectomy. Surgery is palliative for children with diffuse K_ATP_HI, but children with focal K_ATP_HI are cured with a limited pancreatectomy. Therefore, distinguishing between diffuse and focal disease and localizing the focal lesion in the pancreas are crucial aspects of HI management. Since 2003, ^18^ F-DOPA PET scans have been used to differentiate diffuse and focal disease and localize focal lesions with higher sensitivity and specificity than more invasive interventional radiology techniques. Hyperinsulinism remains a challenging disorder, but recent advances in the understanding of its genetic basis and breakthroughs in management should lead to improved outcomes for these children.

## Introduction

Congenital hyperinsulinism (HI) is the most common cause of persistent hypoglycemia in infants and children, which if unrecognized may lead to development delays and permanent neurologic damage**.** In general, the high risk of brain damage appears to be due to delays in diagnosis and treatment rather than a consequence of the genetic defects and, thus, is potentially preventable [1].

Within the last two decades, major advances have been made in understanding the molecular and genetic basis of HI. This work has helped elucidate the physiology of beta cell function and insulin regulation, as well as advanced clinical care for children with HI. Among the nine known genetic causes of HI, mutations in the genes encoding the ATP-sensitive potassium channel represent the most common defect accounting for the majority of the cases [2]. At least 50% of the children carrying these mutations have a focal form of the disease and can be cured by surgery, thanks to the introduction of novel imaging techniques. In this article, we will review the genetic basis of HI with a focus on the recently identified genetic causes and mechanisms of disease, describe the recommended diagnostic work-up and discuss recent advances in the management of this challenging disorder.

### Molecular genetics

**Figure 1:**
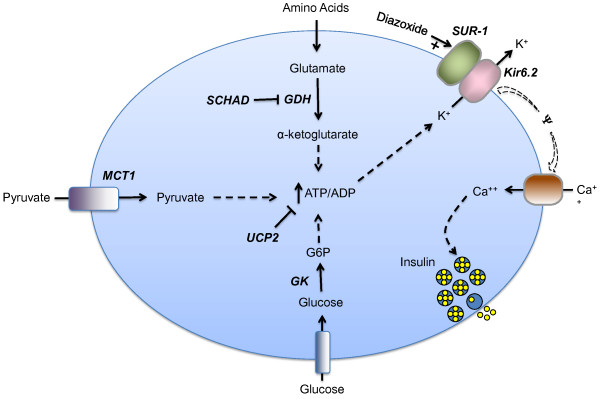
**Genetic defects in the beta cell leading to hyperinsulinism.** In the pancreatic beta cell, ATP production from fuel metabolism leads to inhibition and closure of ATP-sensitive potassium channels, which triggers membrane depolarization and opening of voltage-dependent calcium channels. The resulting increase in cytosolic calcium triggers insulin secretion. Defects in this pathway can result in hyperinsulinism. The known protein defects are depicted in bold italics. Five are inactivating mutations: SUR-1 (sulfonylurea receptor), Kir6.2 (potassium channel), SCHAD (short-chain 3-OH acyl-CoA dehydrogenase), UCP2 (uncoupling protein 2), HNF-4α (hepatic nuclear transcription factor 4α), and HNF-1α (hepatic nuclear transcription factor 1α). The last 2 are transcription factors and are not depicted in the figure. Three are activating mutations: GK (glucokinase), GDH (glutamate dehydrogenase), MCT-1 (monocarboxylate transporter 1). Positive effects are shown by a plus arrow; negative effects by a minus arrow. Dashed arrows denote multiple steps in a pathway. G6P = glucose-6-phosphate, ATP = adenosine triphosphate, ADP = adenosine diphosphate.

Nine genes expressed in the ß-cell have been implicated in the pathophysiology of HI [Figure 1. They include *ABCC8* and *KCNJ11* encoding SUR-1 and Kir6.2, the two subunits of the ATP-sensitive potassium channel (K_ATP_ channel); *GLUD1* encoding glutamate dehydrogenase (GDH); *GCK* encoding glucokinase (GK); *HADH* encoding short-chain 3-hydroxyacyl-CoA (SCHAD); *UCP2* encoding uncoupling protein 2 (UCP2); *HNF4A* and *HNF1A* encoding the transcription factors hepato-cyte nuclear factor 4-alpha (HNF-4α) and hepatocyte nuclear factor 1-alpha (HNF-1α), respectively; and ectopic expression of monocarboxylate transporter 1 (MCT-1) encoded by *SLC16A1.* Many of these genes are also involved in the pathogenesis of monogenic diabetes, including *ABCC8*, *KCNJ11*, *GCK*, *HNF4A*, and *HNF1A*. There remain approximately 50% of diazoxide-responsive cases and 10% of diazoxide-unresponsive cases of HI with unknown genetic etiology, suggesting that additional, yet to be identified genes are implicated in the pathogenesis of HI [3].

#### K_ATP_-hyperinsulinism

Inactivating mutations in the genes encoding the two subunits of the ß-cell ATP-sensitive potassium channel (K_ATP_ channel), *ABCC8* and *KCNJ11* (encoding SUR-1 and Kir6.2, respectively), cause the most common and severe form of hyperinsulinism, although mutations in *ABCC8* are more common [4,5]. The effect of these mutations on channel expression and function determines the clinical phenotype, particularly the response to diazoxide, a K_ATP_ channel opener used in the treatment of HI [2]. Thus, K_ATP_HI can been classified into three subtypes: (1) recessive diazoxide-unresponsive, (2) dominant diazoxide-unresponsive and (3) dominant diazoxide-responsive [6-9]. Clinically, the first two groups are undistinguishable. These children present as neonates with large for gestational age birth weights and severe hypoglycemia that requires high glucose infusion rates and fails to respond to therapy with diazoxide, because there are no functional K_ATP_ channels (recessive defects) or their function is severely impaired (dominant defects) [8]. Therefore, children with recessive or dominant diazoxide-unresponsive K_ATP_HI, frequently require pancreatectomy to control their hypoglycemia. The third group (dominant diazoxide-responsive) typically has less severe hypoglycemia, which may not be noted at birth.

The pathophysiology of K_ATP_HI is characterized by a failure to suppress insulin secretion as glucose concentration falls, manifesting as severe fasting hypoglycemia, and a failure to increase insulin secretion in response to a glucose load [2]. In contrast to the impaired glucose-stimulated insulin secretion, amino acids trigger insulin release in some individuals with K_ATP_HI, causing severe protein-induced hypoglycemia [10].

In the 1980s, it was recognized that some patients with severe hyperinsulinism were cured after a partial pancreatectomy. On histologic inspection, although the majority of their pancreas appeared normal, a focal “tumor-like” area of abnormal beta cell proliferation was identified. In contrast, children who were not cured with surgery had a normal number of beta cells, but these cells showed signs of hyperactivity throughout the pancreas [11]. These findings lead to the recognition of two distinct histologic forms of HI: diffuse and focal. The molecular mechanism responsible for these focal adenomatous lesions in which abnormal beta cell proliferation occurs in a discrete region of the pancreas was later described [12]. The pathophysiology of focal K_ATP_HI includes a “two hits” mechanism: first, a paternally inherited recessive mutation in *ABCC8* or *KCNJ11*; and second, a deletion of the maternally inherited 11p15 chromosomal region, compensated by paternal uniparental disomy [13]. The loss of maternally expressed genes involved in tumor suppression explains the histological findings of focal K_ATP_HI [12,14].

Clinically, children with the focal form are indistinguishable from those with the recessive diffuse form, presenting with severe hypoglycemia and high glucose requirements. Unlike the diffuse form, focal K_ATP_HI may be cured with surgical resection of the discrete lesion. Thus, the recognition of these cases prior to surgery is critical as explained later.

Recently, a novel “atypical” form of focal hyperinsulinism consisting of morphologic mosaicism of pancreatic islets has been reported, which similar to focal lesions, involves only a portion of the pancreas and may be cured with partial pancreatectomy [15]. The histology in this form shows co-existence of two abnormal islet types (large islets with occasional enlarged nuclei and shrunken islets with small nuclei) in a limited region of the pancreas. No mutations in *ABCC8*, *KCNJ11* or *GCK* were identified in these cases and the molecular mechanism is unknown at this time.

#### GDH-hyperinsulinism

The second most common form of HI is due to activating mutations of glutamate dehydrogenase (GDH), encoded by *GLUD1*, leading to the hyperinsulinism/hyperammonemia (HI/HA) syndrome [16]. In the beta cell, GDH is involved in amino acid-stimulated insulin secretion and loss of inhibitory control of GDH in HI/HA leads to dysregulated insulin secretion. The majority of mutations in *GLUD1* occur de novo (70%) with the reminder inherited in an autosomal dominant pattern [17]. Individuals with HI/HA have fasting and protein-induced hypoglycemia, which is easily controlled with diazoxide. Ammonia levels are typically elevated 3–5 times the normal range, but these individuals do not exhibit the classical symptoms associated with hyperammonia due to other causes. Children with HI/HA have increased rates of seizures, most commonly atypical absence, and learning disabilities [18]. These neurologic abnormalities appear to be unrelated to hypoglycemia or elevated ammonia levels.

#### GK-hyperinsulinism

Activating mutations in glucokinase (GK) cause an autosomal dominant form of HI. Glucokinase, encoded by *GCK*, is the “glucose sensor” of the beta cell, triggering insulin secretion in response to rising glucose concentration [19]. Activating mutations lower the threshold for insulin release; thus, the glucose set point for these individuals is lower. GK-HI presents with fasting hypoglycemia of variable degrees of severity and, although the initial reported case responded well to diazoxide, less than a third of the reported cases have been treated successfully with diazoxide [2,20].

#### SCHAD-hyperinsulinism

Short-chain 3-hydroxyacyl-CoA (SCHAD) deficiency leads to an autosomal recessive form of HI. SCHAD, encoded by *HADH*, catalyzes a step in the fatty acid oxidation (FAO) cycle [21]. Although FAO defects are well known to cause hypoglycemia, the connection between an enzyme in the FAO cycle and HI was unclear. Subsequently, it was shown that SCHAD is an inhibitory regulator of GDH, the enzyme involved in amino-acid stimulated insulin secretion, and loss of GDH’s inhibition due to SCHAD deficiency results in insulin dysregulation [22]. Children with SCHAD-HI have fasting and protein-induced hypoglycemia and similar to patients with HI/HA, they respond well to diazoxide therapy. Biochemical markers of SCHAD-HI include increased concentration of 3-hydroxybutyrylcarnitine in plasma and 3-hydroxyglutaric acid in urine. These children do not exhibit the cardiac, skeletal or hepatic dysfunction associated with FAO disorders.

#### UCP2-Hyperinsulinism

Uncoupling protein 2 (UCP2), a membranous mitochondrial carrier, acts as a negative regulator of insulin secretion in the beta cells. Recently, loss of function mutations in UCP2 have been described that result in hyperinsulinism [23]. UCP2 mutations are inherited in an autosomal dominant manner and have been identified in children responding to diazoxide. The reported cases have resolution of HI by 7 months to 6 years of age, which suggests that UCP2-HI is a transient disorder [2].

#### HNF4A and HNF1A-hyperinsulinism

Hepatocyte nuclear factor 4-alpha (HNF-4α), a transcription factor involved in pancreatic development and function, has been classically linked to a monogenic form of early-onset diabetes, MODY1 [24]. Mutations in *HNF4A*, which encodes HNF-4α, have an autosomal dominant pattern of inheritance. A study of families with MODY1 found that carriers of *HNF4A* mutations were born macrosomic and 8 carriers had transient neonatal hypoglycemia with hyperinsulinism identified in 3 of the 8 [25]. Further studies demonstrated *HNF4A* mutations in children presenting with diazoxide-responsive HI [26]. Patients with *HNF4A* mutations respond well to diazoxide and the HI resolves within the first year of life in the majority of cases, although cases of persistent HI caused by *HNF4A* mutations have been reported [27]. The phenotype in these cases is complex and may involve the liver and kidney [28]. Recently, mutations in the transcription factor, hepatocyte nuclear factor 1-alpha (HNF-1α), encoded by *HNF1A* and known to cause MODY3, have been shown to also present with hyperinsulinism in infancy [28]. The mechanism by which loss of function mutations in *HNF4A* and *HNF1A* can lead to this dual phenotype with hypoglycemia in early life and diabetes later, has not been elucidated, but likely implies a changing pattern of gene expression regulation by these transcription factors throughout the life of an individual.

#### MCT1-Hyperinsulinism

Aberrant expression of monocarboxylate transporter 1 (MCT-1) leads to a very rare and unusual form of hyperinsulinism, which is triggered by exercise. Identified in two Finnish families, exercise-induced hyperinsulinism (EIHI) is characterized by episodes of hypoglycemia associated with elevated insulin levels at the time of anaerobic exercise [29]. Autosomal dominant mutations in the regulatory regions of the *SLC16A1* gene, which encodes MCT-1, have been identified [30]. In normal individuals, MCT-1, a transporter of pyruvate and lactate, is not expressed on beta cells, but in EIHI, mutations in the regulatory regions of *SLC16A1* lead to expression of MCT-1 on beta cells. The presence of MCT-1 allows pyruvate, elevated during anaerobic exercise, to enter the beta cell and through the triggering pathway (K_ATP_-mediated), increase insulin release resulting in hypoglycemia [31]. The degree of hypoglycemia associated with exercise is variable and is only partially responsive to diazoxide.

### Diagnosis

For children presenting with hypoglycemia (plasma glucose < 70 mg/dL), prompt diagnosis and establishment of effective treatment is essential to avoid neurologic sequelae. Clinical clues to the diagnosis of the HI include large for gestational age birth weight and severe, persistent hypoglycemia requiring high glucose infusion rates (> 10 mg/kg/min). However, the clinical phenotype of hyperinsulinism is a spectrum and infants with HI can also present with normal birth weights and lower glucose requirements.

The diagnosis of hyperinsulinism is made based on the critical sample obtained during a spontaneous or provoked hypoglycemic event. The threshold blood glucose to obtain the critical sample by convention is set low at < 50 mg/dL to decrease the likelihood of false positive results. If a diagnostic fast is needed to obtain the critical sample, close monitoring of blood glucose, vital signs and mental status is essential to ensure the patient’s safety. Parental dextrose, as well as all appropriate specimen collection tubes, should be at the bedside prior to the start of the fast. Upon completion of diagnostic testing, blood glucoses should be monitored every 10–15 min until they are reliably above 70 mg/dL.

In addition to obtaining the critical sample, the glycemic response to glucagon should be evaluated [Table 1. A detectable insulin level is inappropriate at the time of hypoglycemia and is consistent with insulin excess. A common pitfall in the diagnosis of HI is that insulin concentration is not always elevated, even at the time of hypoglycemia, thus the diagnosis should be based on other indicators of increased insulin action [32]. Laboratories consistent with excess insulin action include suppressed beta-hydroxybutyrate and free fatty acid concentrations as well as an inappropriate glycemic response to glucagon of 30 mg/dL or more at the time of hypoglycemia [33].

**Table 1:** Criteria for diagnosing hyperinsulinism

Clinical Clues	Large for gestational age
	GIR > 10 mg/kg/min^#^
Lab findings with glucose < 50 mg/dL	↓ Beta-hydroxybutyrate (< 0.6 mM)
	↓ Free fatty acids (< 0.5 mM)
	+/− ↑ Insulin
Glycemic Response to Glucagon*	↑ Glucose > 30 mg/dL

In cases with overgrowth and failure to respond to diazoxide and octreotide, activating mutations in AKT2 should be considered in the differential diagnosis [34]. Beckwith-Wiedemann syndrome, neonatal panhypopituitarism and congenital disorders of glycosylation should also be considered and appropriately evaluated if warranted by clinical features. Beckwith-Wiedemann syndrome has significant clinical heterogeneity and is characterized by hemihypertrophy, macrosomia, macroglossia and predisposition to embryonal tumors. Neonates with panhypopituitarism may have diagnostic findings identical to HI with suppressed ketones and free fatty acids and a glycemic response to glucagon. Clinical features suggestive of panhypopituitarism include midline defects and micropenis. Congenital disorders of glycosylation are a highly variable group of disorders caused by abnormal glycosylation of N-linked oligosaccharides and hypoglycemia may be found with failure to thrive and liver dysfunction. Low growth hormone and cortisol at the time of hypoglycemia are not diagnostic of growth hormone deficiency or adrenal insufficiency and the appropriate stimulation tests should be performed to confirm those diagnoses [35]. Insulinomas should be considered in the differential diagnosis of children presenting with hyperinsulinemic hypoglycemia beyond infancy, particularly during the second decade of life.

**Figure 2:**
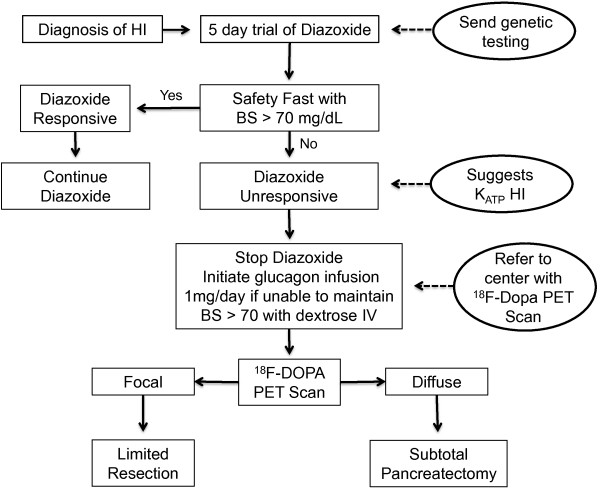
**Algorithm for the treatment of hyperinsulinism.** Assessing the response to diazoxide is a critical step in the management of HI. Patients who fail to respond to diazoxide will most likely have K_ATP_ channel defect and require referral to a specialized center with ^18^ F DOPA PET scan capability. A safety fast should be 8 to 18 h long depending on the age of the patient. Note that octreotide is not recommended as pre-operative treatment in neonates with HI due to high rate of treatment failure and risk of necrotizing enterocolitis. K_ATP_ = ATP-sensitive potassium channel, ^18^ F DOPA PET = 18-fluoro L-3,4-dihydroxyphenylalanine positron emission tomography.

A failure to respond to maximum dose of diazoxide (15 mg/kg/day) after at least 5 days of treatment, suggests a K_ATP_ channel defect as the most likely cause of hyperinsulinism. Such children are potential surgical candidates and require referral to a specialized HI center with 18-fluoro L-3,4-dihydroxyphenylalanine positron emission tomography (^18^ F-DOPA PET) scan availability [Figure 2. Commercial genetic testing is available for the four most common HI genes (*ABCC8*, *KCNJ11*, *GLUD1* and *GCK*) and for the HNFs defects. As a cost-reducing measure, genetic testing should be targeted based on the clinical phenotype; for example sending for *GLUD1* in children who are responsive to diazoxide and have elevated ammonias. Our recommendation is to send genetic testing as soon as possible for the child and his/her parents, especially for the diazoxide-unresponsive cases as the detection of a single recessive paternal K_ATP_ mutation (*ABCC8* or *KCNJ11*) has a positive predictive value of 94% for focal hyperinsulinism [3].

### Management

The therapeutic goal for hyperinsulinism as well as other hypoglycemic disorders is to achieve and maintain plasma glucoses greater than 70 mg/dL. In the 1960s, the antihypertensive, diazoxide with its known side effect of hyperglycemia, was first used to treat hyperinsulinism [36]. The 1970s saw the introduction of octreotide as an HI treatment [37]. These drugs remain the mainstay of medical treatment for HI. Diazoxide acts to open the K_ATP_ channel, decreasing insulin secretion and is the first-line agent for HI, although most cases of K_ATP_HI do not respond.

The therapeutic dose range of diazoxide is wide (5 to 15 mg/kg/day) and varies according to the severity of the phenotype. Patients with severe hypoglycemia and high glucose requirements should be started on the maximum dose of diazoxide at 15 mg/kg/day. Patients with more mild disease can be started on doses of 5–10 mg/kg/day, which should be increased if there is no response after several days of treatment. The half-life of diazoxide in children is between 9.5-24 h [38] and is unknown in neonates, leading to controversy as to whether twice a day or three times a day dosing is appropriate. In general, we find that for diazoxide-responsive children, dosing twice daily is sufficient to provide appropriate control. To evaluate diazoxide’s efficacy after 5 days of therapy, a safety fast should be performed with a duration lasting 8 to 18 h based on the age of the patient. Continued hypoglycemia after at least 5 days of the maximum dose (15 mg/kg/day) is considered a treatment failure. The side effects of diazoxide include hypertrichosis and fluid retention. Hypertrichosis is often quite severe, but resolves after stopping the drug. Fluid retention, especially in neonates, may require the use of a diuretic, such as chlorothiazide, but stronger loop diuretics should be avoided.

The second-line agent, octreotide decreases insulin secretion through hyperpolarization of the beta cells and inhibition of calcium channels. Octreotide is associated with frequent treatment failure due to the development of tachyphylaxis. More importantly, octreotide has recently been associated with the occurrence of fatal necrotizing enterocolitis and therefore, should be used with caution in neonates [39]. Our center no longer recommends its use in neonates pre-operatively. It continues to be used post-operatively in children with diffuse disease who remain hypoglycemic following subtotal pancreatectomy. Successful treatment with long-acting formulations of octreotide has recently been reported [40,41].

Glucagon can be used as a continuous intravenous infusion of 1 mg/day to lower glucose infusion rate requirements in infants awaiting surgery. Trials of glucagon as a subcutaneous infusion through a pump were largely unsuccessful due to the drug’s lack of stability in solution [42,43]. A new potential therapeutic approach to children with K_ATP_ defects involves inhibition of GLP-1 action, an incretin known to increase insulin secretion and lower blood glucose. Recently, a GLP-1 receptor antagonist, exendin-(9–39) has been shown to elevate fasting blood glucose in individuals with hyperinsulinism [44].

Surgical intervention is indicated in children who have a focal lesion that can be cured with resection and in children with diffuse disease who fail medical therapy. Pursuing surgery in the latter group requires a careful consideration of risks and benefits. The benefit of surgery in this group is that their hypoglycemia is often easier to manage following a pancreatectomy, but this must be weighed against the risks of a surgical procedure and long-term complications, such as diabetes. Those children with diffuse disease who have very limited fasting tolerances (less than 2–3 h) and very high glucose infusion requirements will most likely require a pancreatectomy. However, some children with diffuse disease and longer fasting tolerances (6–8 h) may be managed with a combination of frequent feeds, enteral dextrose and/or octreotide. The risks of this management approach include potentially more frequent hypoglycemia and exposure to octreotide.

The largest advance in the management of children with HI over the past decade was the introduction of imaging with ^18^ F-DOPA PET to differentiate focal from diffuse disease and to localize focal lesions [45]. As discussed earlier, children with focal K_ATP_HI can be cured with surgical resection of the lesion. In contrast, for children with diffuse HI, surgery is palliative. Differentiating focal from diffuse disease and accurately identifying the location of focal lesions in the pancreas is crucial for ensuring that children with focal K_ATP_HI are successfully cured. Conventional imaging techniques, such as CT or MRI, cannot identify focal lesions and interventional radiology techniques, such as transhepatic portal venous sampling or arterial calcium stimulation are highly invasive and have poor accuracy for differentiating diffuse from focal HI and for localizing the focal lesion [46].

**Figure 3:**
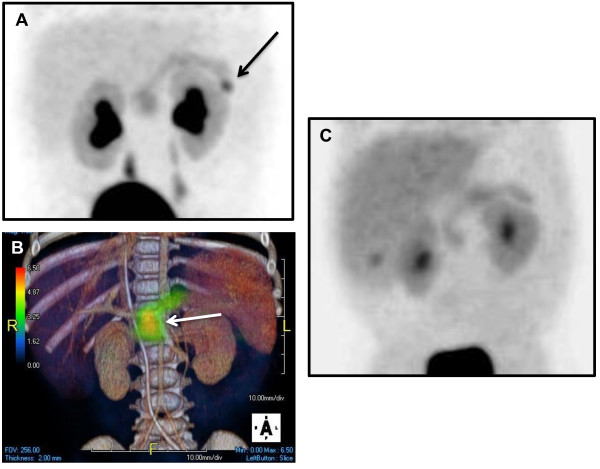
**A. Frontal view of a 3D maximum intensity projection (MIP) 18-fluoro L-3,4-dihydroxyphenylalanine positron emission tomography (**^**18 **^**F-DOPA PET) image demonstrating a focal lesion in the tail of the pancreas (arrow). B**. A frontal view 3D MIP ^18^ F-DOPA PET image fused with a contrast-enhanced CT shows a focal lesion in the pancreatic head (white arrow). **C**. Frontal view of a 3D MIP showing non-uniform pattern of uptake with increased activity throughout the pancreas consistent with diffuse disease. Note the normal liver, kidney and bladder uptake.

The uptake of ^18^ F-DOPA identifies neuroendocrine tissue, which takes up amino acid precursors of dopamine, including DOPA. In diffuse HI, the uptake of the tracer is uniform throughout the pancreas; in contrast, a focal lesion will have greater uptake in a specific region compared to the surrounding tissue [Figure 3. Since 2003, ^18^ F-DOPA PET scans have been used to differentiate diffuse from focal HI and to localize focal lesions prior to surgery [47]. In the largest series to date of 50 patients who underwent ^18^ F-DOPA PET scans, followed by surgery, the sensitivity for diagnosing focal disease was 75% and the location of the focal was correctly identified in 100% of cases [45]. Similar results have been reported in smaller series [48,49]. A meta-analysis in 2012 showed superiority of the ^18^ F-DOPA PET scan compared to interventional radiology techniques for diagnosing and localizing focal lesions [50].

Accurate localization of the focal lesion aids in pre-operative planning and in select cases (lesions on the anterior surface of the body and tail), allows for the use of laparoscopic techniques [2]. Intraoperative biopsies and frozen section evaluation by experienced pathologists allows for confirmation of a focal lesion and guides the extent of pancreatic resection. Children with diffuse HI who fail medical management will require a subtotal pancreatectomy and placement of a gastrostomy tube to help with the post-operative management since most of these children continue to have hypoglycemia, although less severe [51].

Of the surgical cases performed at the Children’s Hospital of Philadelphia, 95% of patients with focal disease have been cured and the majority required less than a 50% pancreatectomy. In contrast, the majority of patients with diffuse disease post-operatively required continued intervention to maintain euglycemia. However, after surgery, their HI can be more easily medically managed. For children with continued hypoglycemia, octreotide during the day combined with continuous intragastric dextrose overnight is effective at preventing octreotide tachyphylaxis and allows for stable glucose control. For the smaller subset of patients with hyperglycemia following subtotal pancreatectomy, insulin may be necessary. The long-term risk of developing diabetes in children with diffuse disease depends on the extent of pancreatic resection [52]. In the largest published series, 91% of children who had undergone a near-total pancreatectomy in infancy required insulin therapy for diabetes by the age of 14 years [53].

## Conclusions

Congenital hyperinsulinism is one of the most complicated and challenging disorders faced by pediatric endocrinologists. The potential for preventing permanent brain damage caused by persistent hypoglycemia, makes it extremely important to identify and treat these children early. The past two decades has seen tremendous progress in understanding the genetic and molecular basis of HI. This understanding has in turn lead to advancements in management and improved outcomes, particularly through the use of ^18^ F-DOPA PET scan to identify and cure focal lesions.

## Abbreviations

HI: Hyperinsulinism; K_ATP_: ATP-sensitive potassium channel; GDH: Glutamate dehydrogenase; HI/HA: Hyperinsulinism/hyperammonemia syndrome; GK: Glucokinase; SCHAD: Short-chain 3-hydroxyacyl-CoA; FAO: Fatty acid oxidation disorder; UCP2: Uncoupling protein 2; HNF-4α: Hepatocyte nuclear factor 4-alpha; HNF-1α: Hepatocyte nuclear factor 1-alpha; MCT1: Monocarboxylate transporter 1; EIHI: Exercise-induced hyperinsulinism; F-DOPA PET: 18-fluoro L-3,4-dihydroxyphenylalanine positron emission tomography.

## Competing interests

The authors declare no competing interests.

## Authors’ contributions

KL drafted the manuscript and designed the figures. DDDL edited the manuscript and figures. Both authors read and approved the final manuscript.
